# Development of an Interpretable Triage Tool for Colorectal Polyp Risk Stratification Within a Population-Based Screening Program: Machine Learning Approach

**DOI:** 10.2196/89422

**Published:** 2026-09-30

**Authors:** Zhenmiao Ye, Jiajin Li, Yimin Xie, Qian Li, Yilun Huang, Guohua Zhang, Xue Yang

**Affiliations:** 1 Wenzhou Center for Disease Control and Prevention (Wenzhou Health Supervision Institution), Wenzhou, China; 2 Faculty of Medicine, Chinese University of Hong Kong, 5/F, JC School of Public Health and Primary Care, Hong Kong, China (Hong Kong), 852 2252-8412; 3 Department of Psychology, Wenzhou Medical University, Wenzhou, Zhejiang, China

**Keywords:** colorectal polyps, machine learning, prediction model, explainability, triage

## Abstract

**Background:**

Colorectal polyps are a major source of precancerous lesions in colorectal cancer (CRC). In many population-based screening programs, a major challenge is the efficient triage of high-risk individuals for diagnostic colonoscopy amid limited endoscopic resources.

**Objective:**

To enrich current screening frameworks, we aimed to develop an accessible, noninvasive risk stratification tool to serve as a digital triage mechanism for colorectal polyps using machine learning (ML) and routinely collected data in China.

**Methods:**

We conducted a cross-sectional study in Wenzhou, China. A total of 4108 individuals (aged 50‐74 y) who were referred for and accepted colonoscopy following an initial population-based risk assessment (questionnaire and fecal test) between May and November 2021 were included. The dataset was split into training and validation sets, and the synthetic minority oversampling technique (SMOTE) was applied only to the training dataset to address class imbalance. Twenty-one noninvasive predictors (lifestyle, dietary, clinical symptoms, and family history) were selected using the Boruta algorithm and least absolute shrinkage and selection operator (LASSO) regression. Nine ML models were evaluated, with the Shapley Additive Explanations (SHAP) method and local interpretable model–agnostic explanations (LIME) used for model interpretability and feature ranking.

**Results:**

Among the 9 ML algorithms evaluated, XGBoost (Extreme Gradient Boosting) achieved the highest area under the receiver operating characteristic curve of 0.672, while LightGBM (Light Gradient Boosting Machine) was identified as the optimal model for clinical triage due to its superior recall (0.6503), a key metric for minimizing missed lesions in community screenings. SHAP analysis identified current smoking status, sex, and family history of colorectal polyps as the most influential factors. Notably, the model captured significant nonlinear risk thresholds, such as an age of 50 years and a BMI of 25 kg/m^2^, providing a more granular risk profile than traditional linear models.

**Conclusions:**

This study provides a scalable, interpretable triage tool to complement existing 2-step CRC screening protocols. By leveraging only noninvasive variables, the LightGBM model enables prioritized referral for colonoscopy, offering a resource-efficient strategy to optimize CRC prevention in resource-limited settings.

## Introduction

Colorectal cancer (CRC) remains one of the most common and preventable malignancies worldwide. In 2020, it accounted for approximately 1.9 million new cases and over 930,000 deaths globally, with incidence rates continuing to rise in many transitioning countries [1]. Colorectal polyps are abnormal growths arising from the mucosal lining of the colon and rectum; adenomatous and serrated polyps represent the earliest morphologically recognizable precursors in the adenoma-carcinoma sequence [2]. Numerous studies have demonstrated that early detection and removal of these precancerous polyps significantly reduce both CRC incidence and mortality [3,4]. Despite this evidence, CRC incidence and mortality rates have not declined substantially in many regions over recent decades, possibly because most precancerous polyps are asymptomatic and therefore remain undetected until advanced stages are reached.

Numerous approaches have emerged for the early detection of colorectal polyps, such as fecal occult blood testing, urinary metabolomics, and computed tomographic colonography [5-7]. Despite these advances, colonoscopy remains the gold standard for definitive diagnosis [8]. Nevertheless, the invasiveness of colonoscopy, coupled with potential complications including bleeding, perforation, and postprocedural pain, together with its substantial cost, restricts its application as a population-wide screening tool [5,9,10]. Given these limitations, a more practical screening strategy is needed: noninvasive, cost-effective tests for the asymptomatic general population, with colonoscopy reserved for those with positive screening results.

The development of colorectal polyps results from a complex interplay of genetic, metabolic, environmental, and lifestyle factors [11-13]. Traditional predictive models, most commonly logistic regression (LR), often oversimplify this multifactorial etiology because of their assumptions of linearity and feature independence. These constraints of traditional models highlight the need for flexible, data-driven approaches like machine learning (ML) that can capture nonlinear relationships and population-specific risk profiles [14]. In recent years, ML has been extensively applied to the diagnosis of colorectal polyps using colonoscopy images and computed tomographic colonography [15]; however, its potential in optimizing the initial triage stage of population-based screening remains underexplored.

Population-based screening programs, which primarily target middle-aged and older adults, typically follow a 2-step protocol: an initial risk assessment (often via simple questionnaires and fecal tests) followed by referral for colonoscopy. However, these programs frequently face resource constraints and limited endoscopic capacity [16]. A major challenge in this setting is the low “yield” of colonoscopies and the difficulty in prioritizing individuals among the large pool of “high-risk” candidates identified by traditional tools. ML excels at uncovering subtle, nonlinear relationships in multidimensional data, where traditional statistical methods often fall short [17]. Consequently, ML holds great promise for refining these screening frameworks by building more accurate risk stratification tools, thereby providing a digital triage mechanism to pinpoint individuals with the highest probability of harboring polyps for prioritized colonoscopy referral.

This study aimed to develop and assess the performance of ML-based risk prediction models for colorectal polyps to enrich existing population-based screening programs. By using readily available, noninvasive data, we sought to provide a cost-effective strategy to precisely stratify risk within the screened population and guide selective, prioritized referral for colonoscopy. This approach is particularly intended to optimize resource allocation and improve screening efficiency in resource-limited settings.

## Methods

### Data Sources and Study Participants

This study was conducted within a population-based CRC screening program targeting residents aged 50 to 74 years in Zhejiang Province, China [18]. Data were collected from all 12 administrative districts and counties of Wenzhou between May and November 2021. This multicenter recruitment strategy allowed for the inclusion of a diverse study population from both highly urbanized city centers and semiurban and rural regional areas, reflecting a wide spectrum of socioeconomic statuses and lifestyle patterns.

The program followed a systematic 2-step triage protocol. All enrolled residents underwent a primary risk assessment consisting of a simple risk evaluation questionnaire and a fecal immunochemical test. Individuals identified as “high-risk” by the questionnaire or those with a positive fecal immunochemical test result were subsequently referred for colonoscopy. Our study focused on those who underwent the full procedure to ensure diagnostic gold-standard confirmation.

Of the 276,395 individuals initially enrolled in the program, 257,481 did not undergo colonoscopy and were excluded. This high exclusion rate was attributed to several factors, including individuals not being identified as “high-risk” during the initial screening or declining colonoscopy due to anxiety regarding the invasive procedure or logistical inconvenience. Among the remaining 18,914 participants who completed colonoscopy, 12,094 were diagnosed with colorectal polyps, while 6820 did not. Cases were defined as individuals with newly diagnosed, histologically confirmed colorectal polyps who were aged 50 to 74 years and had no history of gastrointestinal surgery, chemotherapy, radiotherapy, or other gut-related treatments. Controls were individuals aged 50 to 74 years with a normal colonoscopy (no colorectal polyps detected). Participants were further excluded if they had a history of any malignancy, precancerous gastrointestinal lesions, hereditary CRC syndromes (eg, Lynch syndrome or familial adenomatous polyposis), inflammatory bowel disease, severe comorbidities (eg, serious psychiatric disorders or advanced cardiopulmonary disease), or incomplete data on key variables. After applying these criteria, the final study dataset consisted of 4108 participants: 816 cases with colorectal polyps and 3292 controls without colorectal polyps.

### Data Extraction

Potential predictors of colorectal polyps were selected based on an extensive literature review, clinical expert consultation, and practical feasibility in population-based screening settings [19,20]. All selected variables could be easily obtained through routine questionnaires and basic physical examinations in daily clinical practice. Data were collected once during the screening program and categorized as follows: (1) sociodemographic and lifestyle factors, including age, sex, education level, marital status, smoking status, alcohol consumption, physical activity, family history of cancer (FHC) and/or family history of colorectal polyps (FHPs), and history of colonoscopy (HCS); (2) comorbidities and medication use, including hypertension, diabetes mellitus, gout, hyperlipidemia, use of nonsteroidal anti-inflammatory drugs, and anticoagulant therapy; (3) clinical symptoms and anthropometric measurements, including unexplained weight loss, hematochezia, abdominal pain, abdominal distension, palpable abdominal mass, abnormal bowel habits, iron-deficiency anemia, BMI, and waist circumference; and (4) dietary habits, including consumption of vegetables, fruits, white meat, red meat, processed meat, pickled foods, fried foods, whole grains, and legumes. Variables were incorporated into the models based on their raw distribution to minimize information loss. Sociodemographic and clinical factors were treated as binary or continuous where appropriate. Notably, adequate physical activity was dichotomized based on the frequency of moderate-to-vigorous intensity exercise, with more than 3 times per week defined as “adequate” and less than 3 times per week as “inadequate.” Secondhand smoking was defined as a binary variable based on self-reported exposure (“yes” or “no”). Medical history, medication use, and symptoms were all dichotomized as “yes” or “no.” Detailed variable definitions and units are summarized in Table 1.

**Table 1.** Baseline characteristics of the included participants^a^.

Characteristics	Entire cohort (n=4108)	Colorectal polyps (n=816, 19.9%)	Controls (n=3292, 80.1%)	*P* value^a^
Sociodemographic factors				
Age (y), median (IQR)	62 (55‐67)	63 (56‐68)	61 (55‐67)	<.001^b^
BMI (kg/m^2^), median (IQR)	23.6 (21.97‐25.48)	23.9 (22.2‐25.8)	23.6 (21.9‐25.4)	<.001^c^
Waist circumference (cm), median (IQR)	84.0 (80‐88)	85 (80‐89)	84 (79‐88)	<.001^c^
Male, n (%)	1799 (43.8)	460 (56.4)	1339 (40.7)	<.001^d^
Adequate physical activities, n (%)	783 (19.1)	163 (20.0)	620 (18.8)	.49^d^
Education attainment (y), n (%)				<.001^d^
<6	3114 (75.8)	587 (71.9)	2527 (76.8)	
6‐12	925 (22.5)	213 (26.1)	712 (21.6)	
>12	69 (1.7)	16 (2.0)	53 (1.6)	
Marital status, n (%)				.46^d^
Coupled	3927 (95.6)	772 (94.6)	3155 (95.8)	
Single or separated	181 (4.4)	44 (5.4)	137 (4.2)	
Smoking status, n (%)				<.001^d^
Current	636 (15.5)	231 (28.3)	405 (12.3)	
Never or former	3472 (84.5)	585 (71.7)	2887 (87.7)	
Secondhand smoking, n (%)	183 (4.5)	51 (6.2)	132 (4.0)	<.001^d^
Alcohol consumption, n (%)				<.001^d^
Current	633 (15.4)	170 (20.8)	463 (14.1)	
Never or former	3475 (84.6)	646 (79.2)	2829 (85.9)	
Family history of cancer, n (%)	267 (6.5)	71 (8.7)	196 (6.0)	<.001^d^
Family history of colorectal polyps, n (%)	111 (2.7)	31 (3.8)	80 (2.4)	<.001^d^
History of colonoscopy, n (%)	410 (10.0)	67 (8.2)	343 (10.4)	.07^d^
Comorbidity, n (%)				
Hypertension	1087 (26.5)	226 (27.7)	861 (26.2)	.40^d^
Diabetes	417 (10.2)	108 (13.2)	309 (9.4)	<.001^d^
Gout	28 (0.7)	4 (0.5)	24 (0.7)	.61^d^
Hyperlipidemia	201 (4.9)	44 (5.4)	157 (4.8)	.52^d^
Medications, n (%)				
NSAIDs^e^	49 (1.2)	11 (1.3)	38 (1.2)	.78^d^
Anticoagulant	6 (0.1)	1 (0.1)	5 (0.2)	—^f^
Symptoms, n (%)				
Unexplained weight loss	7 (0.2)	7 (0.9)	0 (0)	.40^d^
Hematochezia	21 (0.5)	2 (0.2)	19 (0.6)	.36^d^
Abdominal pain	144 (3.5)	31 (3.8)	113 (3.4)	.69^d^
Abdominal distension	220 (5.4)	45 (5.5)	175 (5.3)	.89^d^
Abdominal mass	5 (0.1)	1 (0.1)	4 (0.1)	—
Abnormal defecation	767 (18.7)	160 (19.6)	607 (18.4)	.47^d^
Iron deficiency anemia	3 (0.1)	0 (0)	3 (0.1)	.89^d^
Dietary intake				
Vegetables, n (%)				.54^d^
Never or 1‐3 days/month	140 (3.4)	30 (3.7)	110 (3.3)	
1‐3 days/week	581 (14.1)	124 (15.2)	457 (13.9)	
4‐6 days/week or every day	3387 (82.5)	662 (81.1)	2725 (82.8)	
Fruits, n (%)				.38^d^
Never or 1‐3 days/month	766 (18.7)	164 (20.1)	602 (18.3)	
1‐3 days/week	1484 (36.1)	298 (36.5)	1186 (36.0)	
4‐6 days/week or every day	1858 (45.2)	354 (43.4)	1504 (45.7)	
Legume, n (%)				.92^d^
Never or 1‐3 days/month	1657 (40.3)	331 (40.6)	1326 (40.3)	
1‐3 days/week	1696 (41.3)	332 (40.7)	1364 (41.4)	
4‐6 days/week or every day	755 (18.4)	153 (18.7)	602 (18.3)	
Whole grain, n (%)				.49^d^
Never or 1‐3 days/month	2015 (49.1)	385 (47.2)	1630 (49.5)	
1‐3 days/week	1154 (28.1)	238 (29.2)	916 (27.8)	
4‐6 days/week or every day	939 (22.8)	193 (23.6)	746 (22.7)	
Processed meat, n (%)				.79^d^
Never or 1‐3 days/month	3209 (78.1)	631 (77.2)	2578 (78.3)	
1‐3 days/week	712 (17.3)	145 (17.8)	567 (17.2)	
4‐6 days/week or every day	187 (4.6)	40 (5)	147 (4.5)	
Red meat, n (%)				.88^d^
Never or 1‐3 days/month	535 (13.0)	102 (12.5)	433 (13.2)	
1‐3 days/week	1668 (40.6)	332 (40.7)	1336 (40.6)	
4‐6 days/week or every day	1905 (46.4)	382 (46.8)	1523 (46.2)	
White meat, n (%)				.22^d^
Never or 1‐3 days/month	812 (19.8)	149 (18.3)	663 (20.1)	
1‐3 days/week	1629 (39.7)	344 (42.2)	1285 (39.1)	
4‐6 days/week or every day	1667 (40.5)	323 (39.5)	1344 (40.8)	
Pickled food, n (%)				.20^d^
Never or 1‐3 days/month	2723 (66.2)	520 (63.7)	2200 (66.8)	
1‐3 days/week	1062 (25.9)	234 (28.7)	831 (25.3)	
4‐6 days/week or every day	323 (7.9)	62 (7.6)	261 (7.9)	
Fried food, n (%)				.41^d^
Never or 1‐3 days/month	3672 (89.4)	720 (88.2)	2952 (89.6)	
1‐3 days/week	354 (8.6)	76 (9.3)	278 (8.4)	
4‐6 days/week or every day	82 (2)	20 (2.5)	62 (2)	

a *P*<.001 indicates a significant difference between the colorectal polyps group and the control group.

b Comparison of median values between the colorectal polyps group and the control group using the Wilcoxon rank-sum test.

c Comparison of the mean values between the colorectal polyps group and the control group using Student *t* test.

d Comparison of percentages between the colorectal polyps group and the control group using the chi-square test.

e NSAID: nonsteroidal anti-inflammatory drug.

f Not applicable.

### Data Preparation

Several issues were present in the raw data, including missing values, outliers, and class imbalance, all of which could substantially affect the construction and performance of colorectal polyp risk prediction models. Missing values were rare, ranging from 0% to 3.5% across all variables. To ensure the appropriateness of our handling method, the Little missing completely at random test was conducted, which yielded a nonsignificant result (*P*=.37), indicating that the data were missing completely at random. Consequently, we performed complete-case analysis by excluding any records with missing values. Outliers were defined as values greater than or less than 1.5 times the IQR beyond the upper or lower quartile. We replaced these outliers with the 1st or 99th quantile values, thereby ensuring that extreme outliers did not disproportionately influence feature selection and model training, particularly for regularization-based algorithms [21].

The dataset exhibited marked class imbalance, with the majority of participants having no colorectal polyps, which could bias models toward negative predictions. To address this, we applied the synthetic minority oversampling technique (SMOTE) only to the training dataset after train-test splitting at an 80:20 ratio. SMOTE generates synthetic examples of the minority class (cases) by interpolating between each minority instance and its k-nearest neighbors until class balance is achieved [22]. Because oversampling was performed within the training dataset and conducted on a secure internal network, there was no risk of data leakage.

### Feature Selection

Candidate features for the colorectal polyp risk stratification model exhibited considerable heterogeneity in scale and clinical relevance. To reduce model complexity, enhance training efficiency, and improve interpretability, a systematic feature-selection pipeline was implemented. Relevant clinical experts were first consulted to prioritize variables with established or plausible associations with colorectal polyps. All subsequent feature-selection procedures were conducted exclusively on the training dataset after splitting. Subsequently, 2 complementary data-driven methods were applied: (1) least absolute shrinkage and selection operator (LASSO) regression embedded within a 10-fold cross-validation framework, which shrinks coefficients of irrelevant predictors to exactly zero through L1 regularization [15], and (2) the Boruta algorithm, a random forest (RF)–based wrapper that identifies truly important features by comparing their importance (*z*-scores) against iteratively generated shadow features, effectively capturing nonlinear relationships and interactions [23]. Features confirmed as important by either LASSO or Boruta were then evaluated for multicollinearity using Spearman correlation analysis; when the absolute correlation coefficient exceeded 0.8, only the variable with greater clinical interpretability or a stronger univariate association was retained.

### Construction of a Colorectal Polyp Risk Stratification Model

Nine well-established ML algorithms were used to develop the colorectal polyp risk stratification models. LR applies the logistic function for binary classification but can overfit with high-dimensional data [24]. Support vector machine constructs optimal separating hyperplanes by maximizing the margin between classes [25]. Gaussian naive Bayes relies on Bayes’ theorem with the assumption of conditional independence among features. The multilayer perceptron addresses linearly inseparable problems by stacking multiple layers of neurons with nonlinear activation functions [26]. Decision tree recursively partitions the feature space, with internal nodes representing feature-based decisions and leaf nodes denoting final class predictions [27]. RF, Extreme Gradient Boosting (XGBoost), Light Gradient Boosting Machine (LightGBM), and categorical boosting (CatBoost) are ensemble methods that aggregate numerous decision trees. While RF uses bagging with random feature selection, the boosting-based algorithms (XGBoost, LightGBM, and CatBoost) use sequential gradient boosting with advanced regularization. Specifically, LightGBM uses gradient-based 1-side sampling and exclusive feature bundling to accelerate training and handle high-dimensional screening data, whereas CatBoost offers native handling of categorical variables, making these ensemble approaches collectively highly effective in complex medical prediction tasks [28]. To optimize hyperparameters and prevent overfitting, 10-fold cross-validation was performed on the training dataset, using 9 folds for training and 1 fold for validation in each iteration.

### Performance Assessment of a Colorectal Polyp Risk Stratification Model

The independent validation dataset, comprising 20% of the total dataset, was used for final model performance evaluation. Models were assessed across 3 key dimensions: discrimination, calibration, and clinical utility. Discrimination was evaluated using accuracy, sensitivity (recall), specificity, positive predictive value, negative predictive value, *F*_1_-score, and Brier score. The area under the receiver operating characteristic curve was calculated for each model, with 95% CIs estimated using the DeLong method. To further evaluate model performance under class imbalance, precision-recall curves were generated, and the average precision (AP) was calculated. AP provides a more robust measure of a model’s ability to identify true positive cases by summarizing the trade-off between precision and recall across various thresholds. Calibration performance was examined through calibration plots and quantified using Brier scores. Clinical usefulness and net benefit across a range of risk thresholds were assessed via decision curve analysis. This comprehensive evaluation framework ensured a robust assessment of both statistical performance and real-world clinical applicability [29].

### Feature Importance and Results Interpretation

ML models are frequently criticized as “black boxes” owing to their complexity and lack of transparency. To address this limitation and enhance clinical trust, we applied the Shapley Additive Explanations (SHAP) framework to the best-performing model [30]. SHAP, grounded in cooperative game theory, provides consistent and locally accurate feature-attribution values that can be aggregated for both global and local interpretability for virtually any ML algorithm. Global model behavior was elucidated through SHAP summary plots, which rank features by their overall impact on model output and illustrate the direction and magnitude of each feature’s effect across the entire dataset. Local interpretability for individual predictions was complemented by local interpretable model–agnostic explanations (LIME), which approximates the complex model locally with an interpretable surrogate to explain specific predictions. Additionally, SHAP dependence plots for the 3 most influential features and those displaying potential nonlinear patterns in the summary plot were generated to visualize interaction effects between each feature and the model’s risk prediction [31].

### Statistical Analysis

Descriptive statistics were presented as mean (SD) or median with IQR for continuous variables, depending on the data distribution, and as frequencies with percentages for categorical variables. To mitigate excessive sparsity, low-frequency categories were merged when clinically appropriate. Between-group comparisons were performed using the 2-tailed Student *t* test, Wilcoxon rank-sum test, or chi-square test, as appropriate. A 2-sided *P*<.05 was considered statistically significant. All data processing, statistical analyses, and ML modeling were conducted using R software (version 4.4.1; R Foundation for Statistical Computing) [32].

### Ethical Considerations

All participants provided written informed consent. The study procedures were carried out in accordance with the Declaration of Helsinki. Ethics approval was obtained from the Ethics Committee of Zhejiang Cancer Hospital (IRB-2023‐464).

## Results

### Baseline Characteristics of Included Participants

A total of 4108 participants were included in the final analysis (816 cases with colorectal polyps and 3292 controls). The participant selection process is illustrated in Figure 1. The baseline characteristics of the study population are summarized in Table 1. The median age of all participants was 62 (IQR 55‐67) years, with 1799 out of 4108 participants (43.8%) being male. In the case group, the median age was 63 (IQR 56‐68) years and 460 out of 816 (56.4%) participants were male, whereas the control group had a median age of 61 (IQR 55‐67) years and 1399 out of 3292 (42.5%) participants were male. Apart from age and sex, participants in the 2 groups were largely comparable across most sociodemographic, clinical, and lifestyle variables. Details of the colorectal polyp composition in the case group are provided in Multimedia Appendix 1.

**Figure 1.**
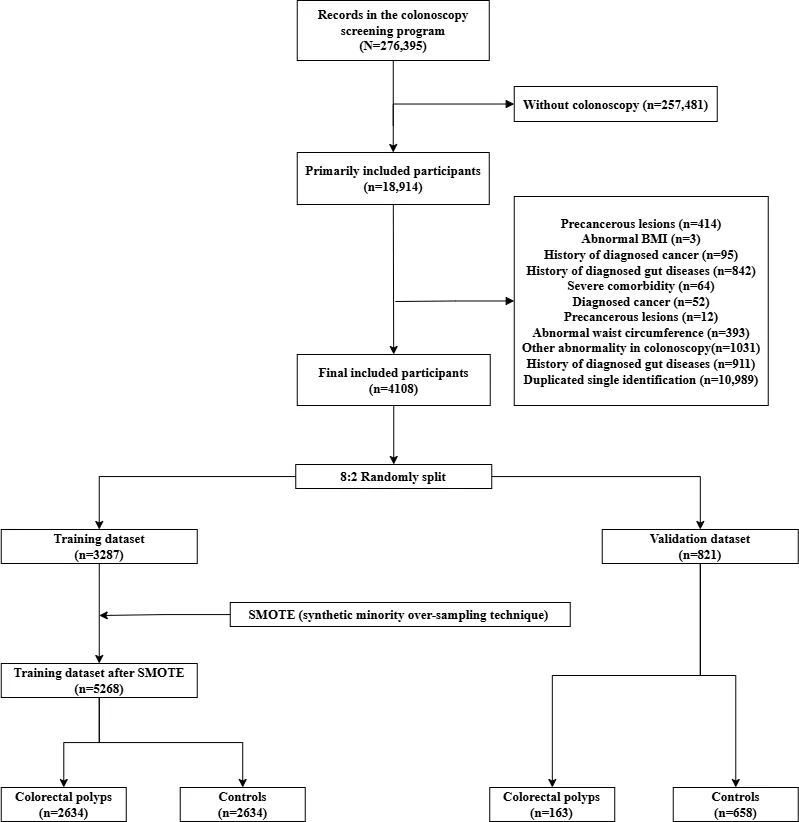
Flowchart of participant selection and data preparation for model construction.

### Feature Selection and SMOTE Implementation

A total of 36 candidate predictors were initially considered based on a literature review and consultation with clinical experts before data splitting. LASSO regression with 10-fold cross-validation was first performed for feature selection and regularization. Using the 1 SE rule, the optimal λ of 0.0046 was selected, retaining 18 informative features with nonzero coefficients (Figure 2 and Multimedia Appendix 2). Independently, the Boruta algorithm confirmed the importance of multiple features and rejected 24 variables as unimportant (Figure 3). Features identified as important by either LASSO or Boruta (n=21) were carried forward. These comprised FHP, FHC, HCS, age, sex, BMI, waist circumference, current smoking, secondhand smoke exposure, alcohol consumption, educational attainment, marital status, intake frequency of fried food, pickled food, white meat, and red meat, as well as gout, diabetes, hypertension, hematochezia, and unexplained weight loss. Spearman correlation analysis among these 21 features revealed no pair with an absolute correlation coefficient greater than 0.8 (Multimedia Appendix 3). Consequently, all 21 variables were retained for the final ML model development and evaluation. After applying SMOTE, the training dataset (case-to-control ratio=653:3287, 19.86% before balancing) was perfectly balanced, with 2643 cases and 2643 controls, respectively (Figure 1).

**Figure 2.**
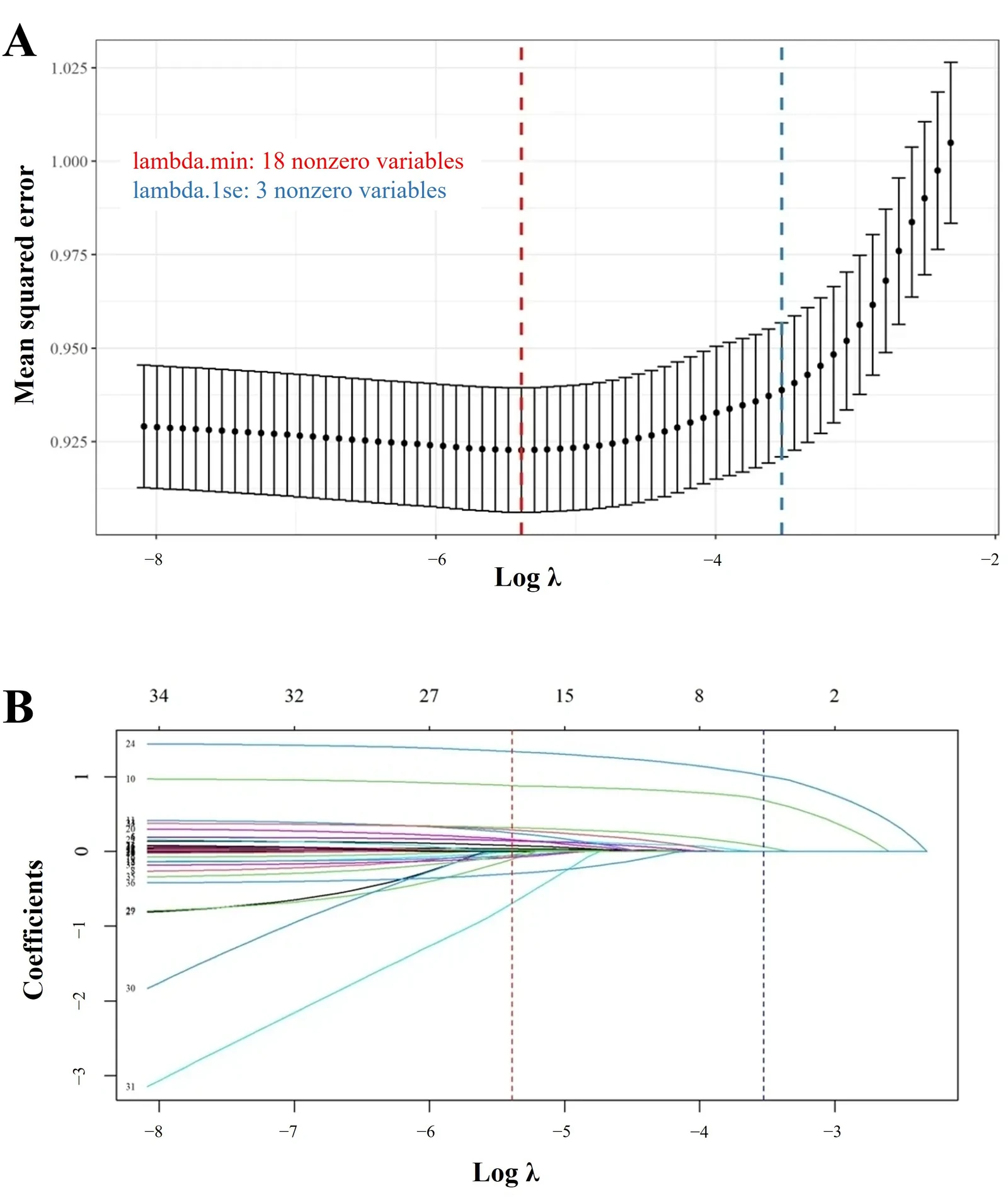
Feature selection using the least absolute shrinkage and selection operator (LASSO) binary logistic regression model. (A) Optimization of the parameter λ in the LASSO model using 10-fold cross-validation under the minimum criterion. (B) LASSO coefficient profiles of 36 variables. The red and blue vertical lines represent the λ values selected through 10-fold cross-validation.

**Figure 3.**
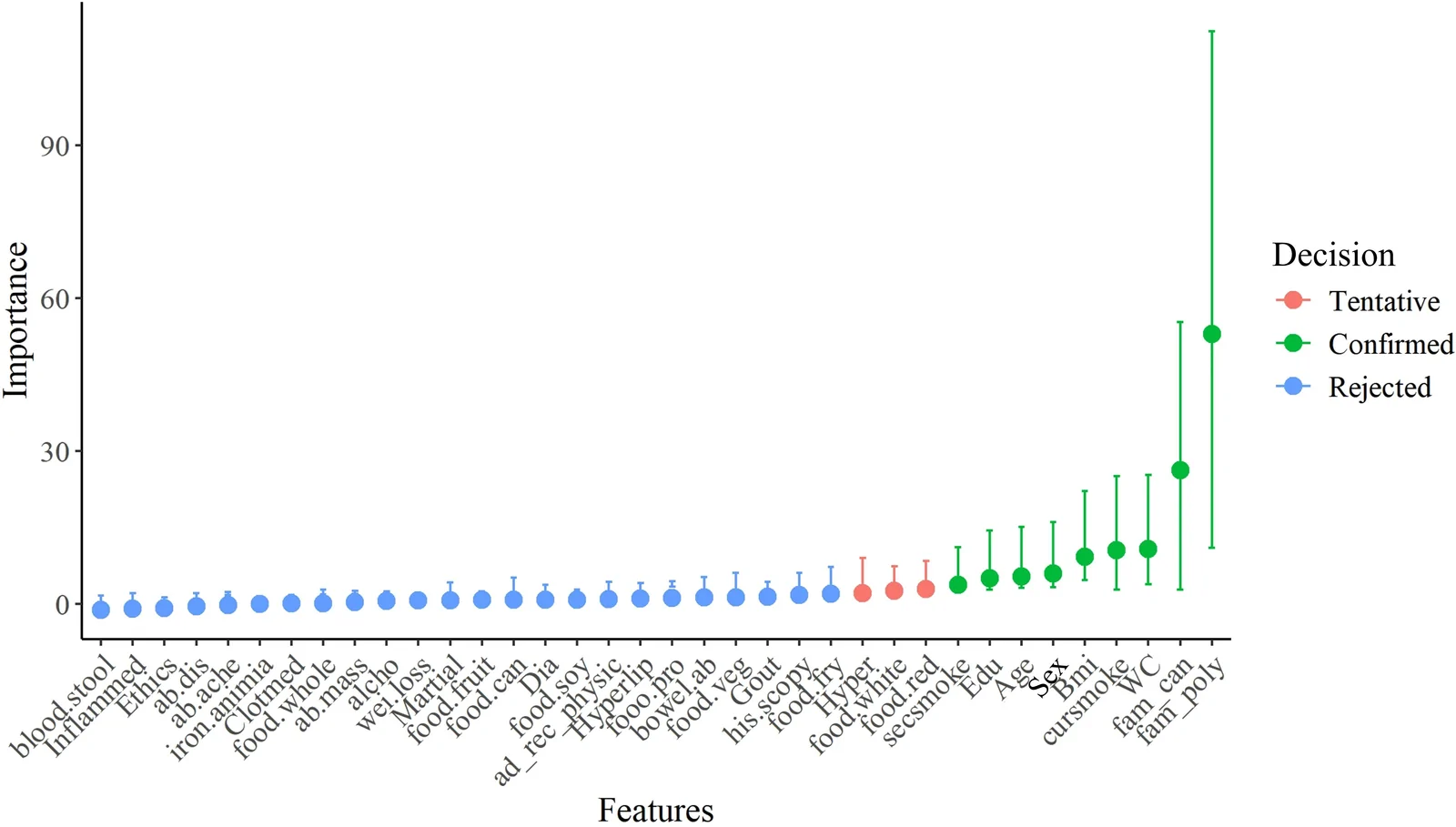
Feature importance based on the Boruta algorithm. The horizontal axis shows the names of the features (full names are provided in Multimedia Appendix 2), and the vertical axis represents the *z*-score of each feature based on the random forest algorithm.

### Comparison of Constructed Prediction Models

The 9 ML models were trained on a balanced dataset and evaluated on an independent validation dataset (n=821). XGBoost and LightGBM emerged as the top-performing models. In Figure 4A to C, XGBoost achieved the highest area under the receiver operating characteristic curve of 0.672 (95% CI 0.623‐0.722) and a higher AP of 0.430 (Multimedia Appendix 4). LightGBM, while having a slightly lower AUC, demonstrated the highest clinical sensitivity, correctly identifying 106 out of 163 cases (Multimedia Appendix 5). This trade-off between precision and recall makes LightGBM particularly valuable for minimizing missed lesions in population-based screening. Decision curve analysis further confirmed that these ensemble models maintained a consistent positive net benefit across a broad range of threshold probabilities (Figure 4D). Calibration performance was assessed via calibration plots (Multimedia Appendix 6). Most models, including XGBoost and LightGBM, exhibited favorable calibration in the low-to-moderate risk range (predicted probability <0.5), with Brier scores consistently below 0.25. While fluctuations were observed in the high-probability zones due to limited sample density, the overall alignment with the ideal diagonal line indicates reliable probability estimates for clinical triage. In conclusion, XGBoost and LightGBM are recommended as the preferred models for clinical risk stratification. The optimal probability cutoffs were derived from the validation dataset. Individuals with risk scores exceeding these thresholds (XGBoost: 0.2432; LightGBM: 0.1395) are recommended for prioritized diagnostic colonoscopy to optimize the detection of precancerous lesions in resource-limited settings.

**Figure 4.**
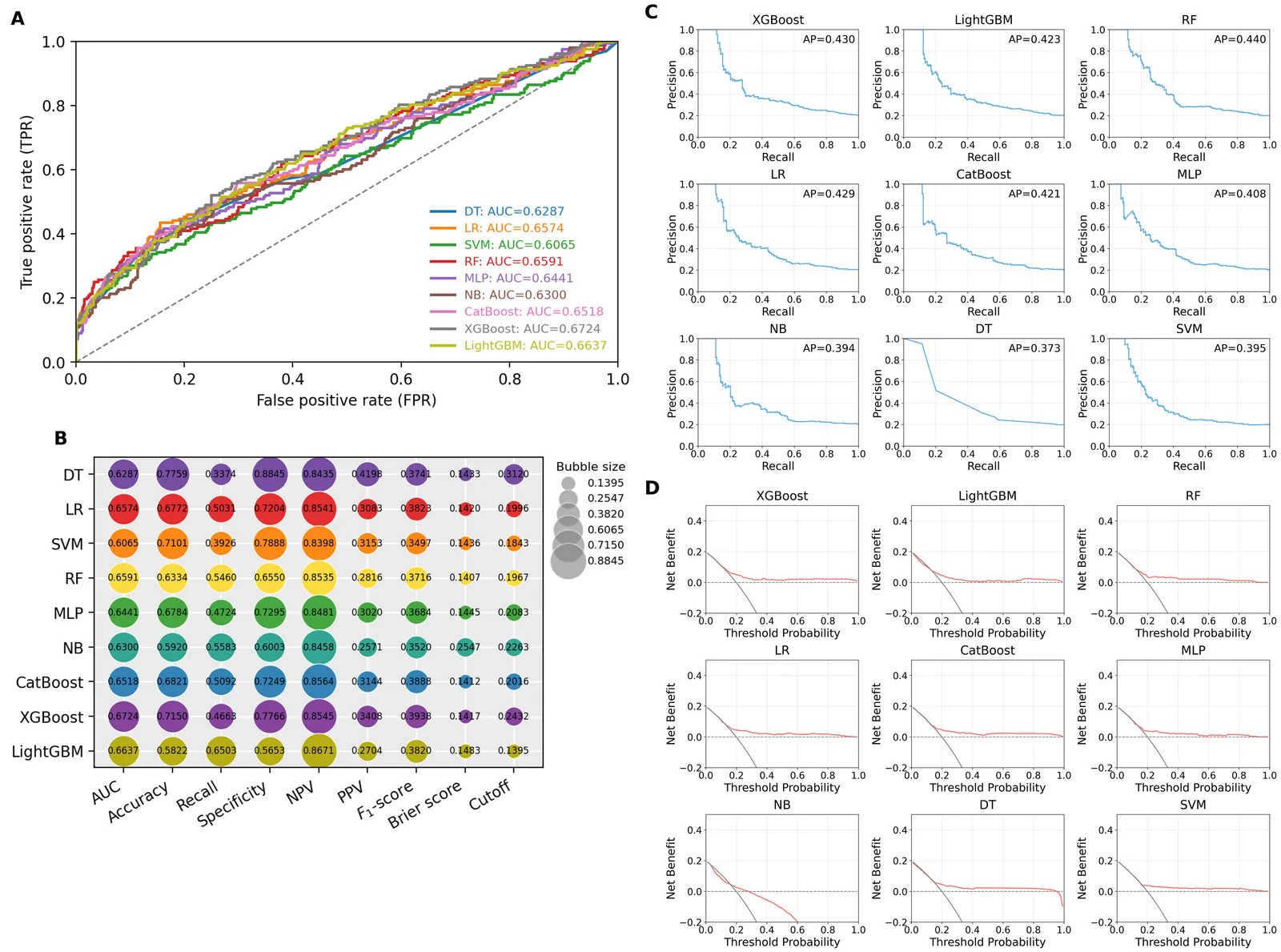
Multidimensional evaluation of machine learning (ML) models for colorectal polyp risk stratification: (A) receiver operating characteristic (ROC) curves; (B) bubble chart comparison of comprehensive performance metrics; (C) precision-recall (PR) curves with average precision (AP) values; a higher area under the PR curve (closer to the top-right corner) indicates superior predictive performance; (D) decision curve analysis (DCA). The red curves represent the ML models; the gray line represents “screen-all,” and the horizontal dashed line represents “screen-none.” Models with higher net benefit (further above the gray and dashed lines) provide greater clinical value for triage. AUC: area under the curve; CatBoost: categorical boosting; DT: decision tree; LightGBM: Light Gradient Boosting Machine; LR: logistic regression; MLP: multilayer perceptron; NB: naive Bayes; RF: random forest; SVM: support vector machine; XGBoost: Extreme Gradient Boosting.

### Feature Importance and Interpretation With the SHAP and LIME Methods

To elucidate the biological and lifestyle-related drivers behind the model’s predictions, we performed global and local interpretability analyses using SHAP and LIME, specifically focusing on the LightGBM model, given its optimal sensitivity for triage screening. Figure 5A presents the global feature importance based on the mean absolute SHAP values. Current smoking status (cursmoke), sex, and a family history of polyps (fam_poly) emerged as the most influential predictors, followed by socioeconomic and anthropometric factors such as education level (Edu) and BMI. The SHAP beeswarm plot (Figure 5B) details the direction and magnitude of these effects across the validation dataset. Notably, the beeswarm distribution for waist circumference, age, and BMI suggested potentially nonlinear relationships with the risk of colorectal polyps, as evidenced by the nonuniform spread of their SHAP values.

**Figure 5.**
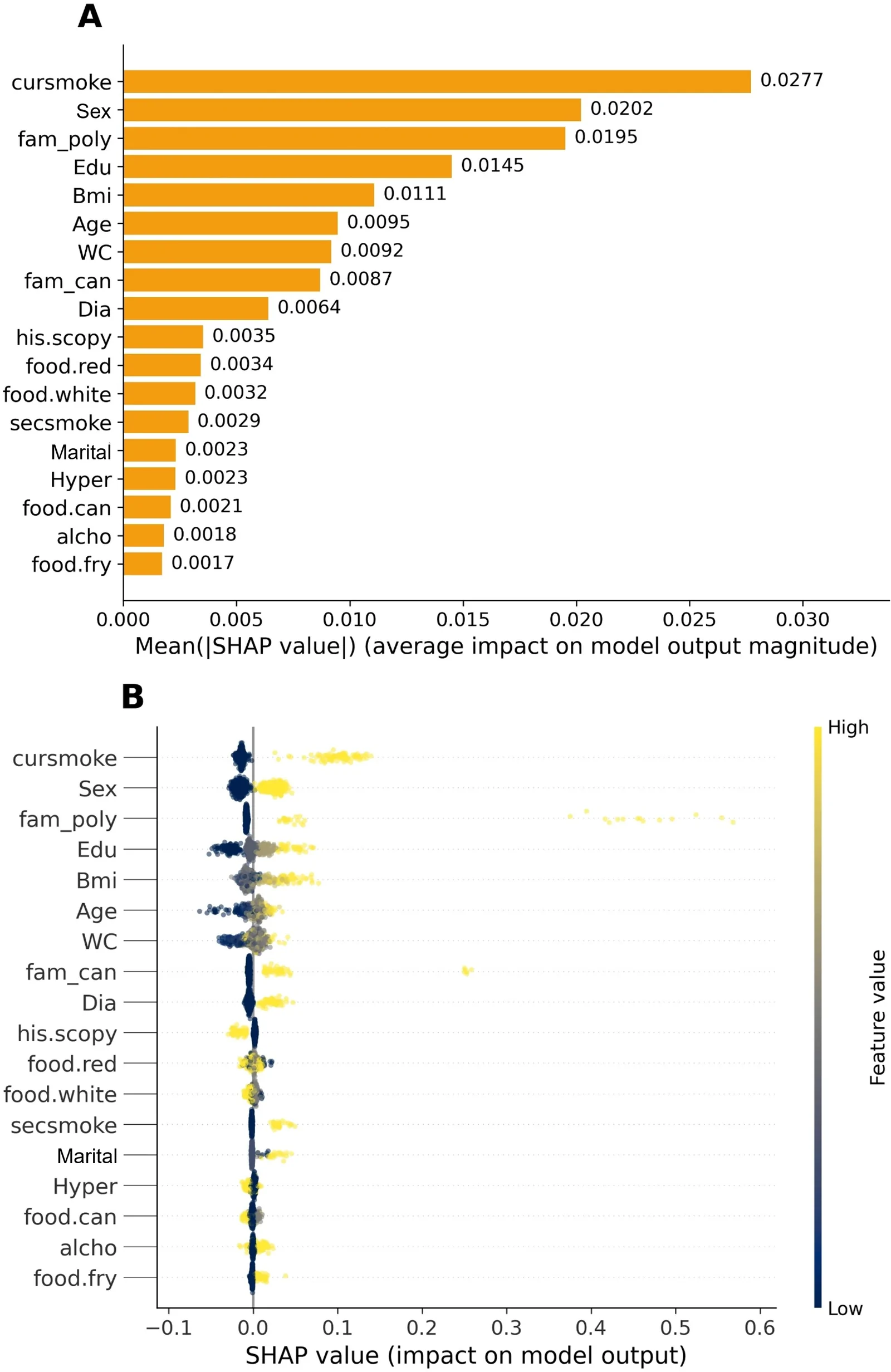
Global feature importance and contribution distribution based on Shapley Additive Explanations (SHAP) method values. (A) Feature importance ranking using a bar chart. A higher mean SHAP value indicates a greater influence on the prediction. (B) SHAP beeswarm plot. Each dot represents an individual in the validation set. Dots to the right of the zero line indicate an increased risk, while those to the left indicate a protective effect. WC: waist circumference.

To further dissect the specific impact of key predictors on colorectal polyp risk, SHAP partial dependence plots were generated for 6 prioritized variables (Figure 6). These plots illustrate the nonlinear trajectories and threshold effects captured by the LightGBM model. For continuous variables, waist circumference exhibited a distinct inflection point at approximately 85 cm, beyond which the SHAP values decreased sharply and nearly linearly (Figure 6A). Similarly, age demonstrated a clear threshold effect at 50 years; while the risk remained below the baseline for younger participants, it increased monotonically thereafter, reinforcing the clinical rationale for intensified screening in the 50 years and older age group (Figure 6C). BMI showed a comparable trend, with the contribution to risk transitioning from protective to causative as BMI exceeded the 25 kg/m² threshold (Figure 6E). Regarding categorical factors, the model captured consistently elevated risks for males (Figure 6F) and current smokers (Figure 6B). Notably, family history of polyps showed the highest magnitude of impact among all categorical features, with a positive history contributing the strongest positive deviation in predicted risk scores (Figure 6D).

**Figure 6.**
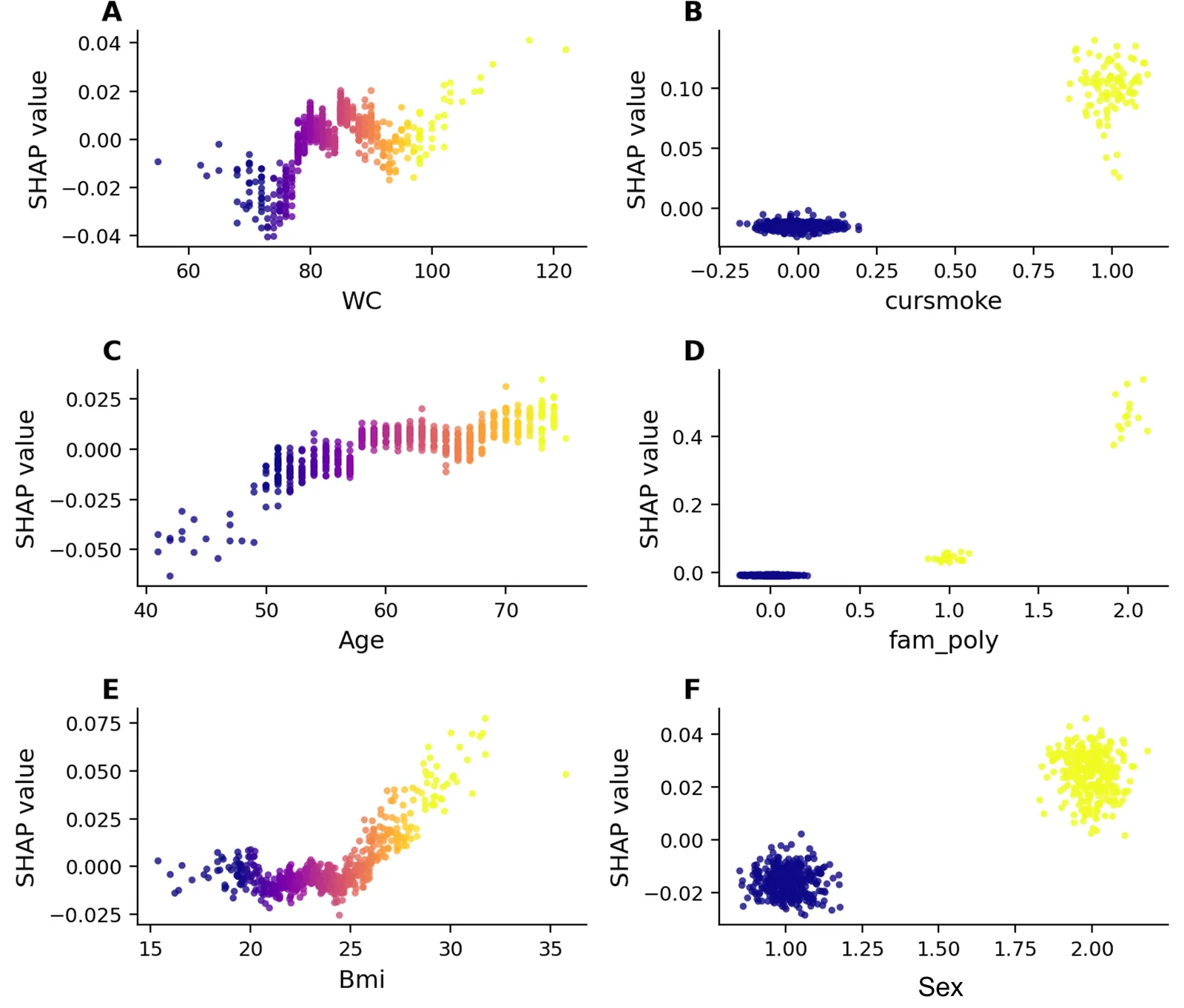
Shapley Additive Explanations (SHAP) method partial dependence plots for key predictors of colorectal polyps. (A,C,E) Continuous variables (waist circumference, age, and BMI); (B,D,F) Categorical and binary variables (current smoking status, family history of polyps, and sex).

To visualize the “push and pull” of individual risk factors, we used SHAP force plots to decompose the prediction process for representative participants (Figure 7A and B). For a low-risk individual (Figure 7A), the final prediction (*f*[*x*]=0.143) was driven below the overall baseline (*E*[*f*(*x*)]=0.198) primarily by the absence of family history (fam_poly=0.0) and nonsmoking status (cursmoke=0.0), which successfully counteracted the risk elevation from diabetes (Dia=1.0). In contrast, for a high-risk individual (Figure 7B), advanced age (69 y) and family history (fam_poly=1.0) served as the dominant forces pushing the risk score toward a positive diagnosis. These visualizations demonstrate how LightGBM integrates disparate clinical signals into a unified risk score.

We further used LIME to validate the model’s decision-making logic through localized linear approximations (Figure 7C and D). For Case 93 (Figure 7C), the model achieved a high-confidence prediction (probability=0.93) for the control, citing the absence of family history as the primary supportive evidence, while identifying the absence of colonoscopy as the only contradictory evidence. Conversely, for Case 63 (Figure 7D), despite certain lifestyle factors acting as contradictory evidence (eg, nonsmoking), the synergistic weight of family history of colorectal polyps (fam_poly=1.0) and cancer (fam_can=1.0) was sufficient to trigger a high-risk alert (probability=0.93). The consistent alignment between SHAP’s global-to-local logic and LIME’s local attribution enhances the clinical reliability of the model.

**Figure 7.**
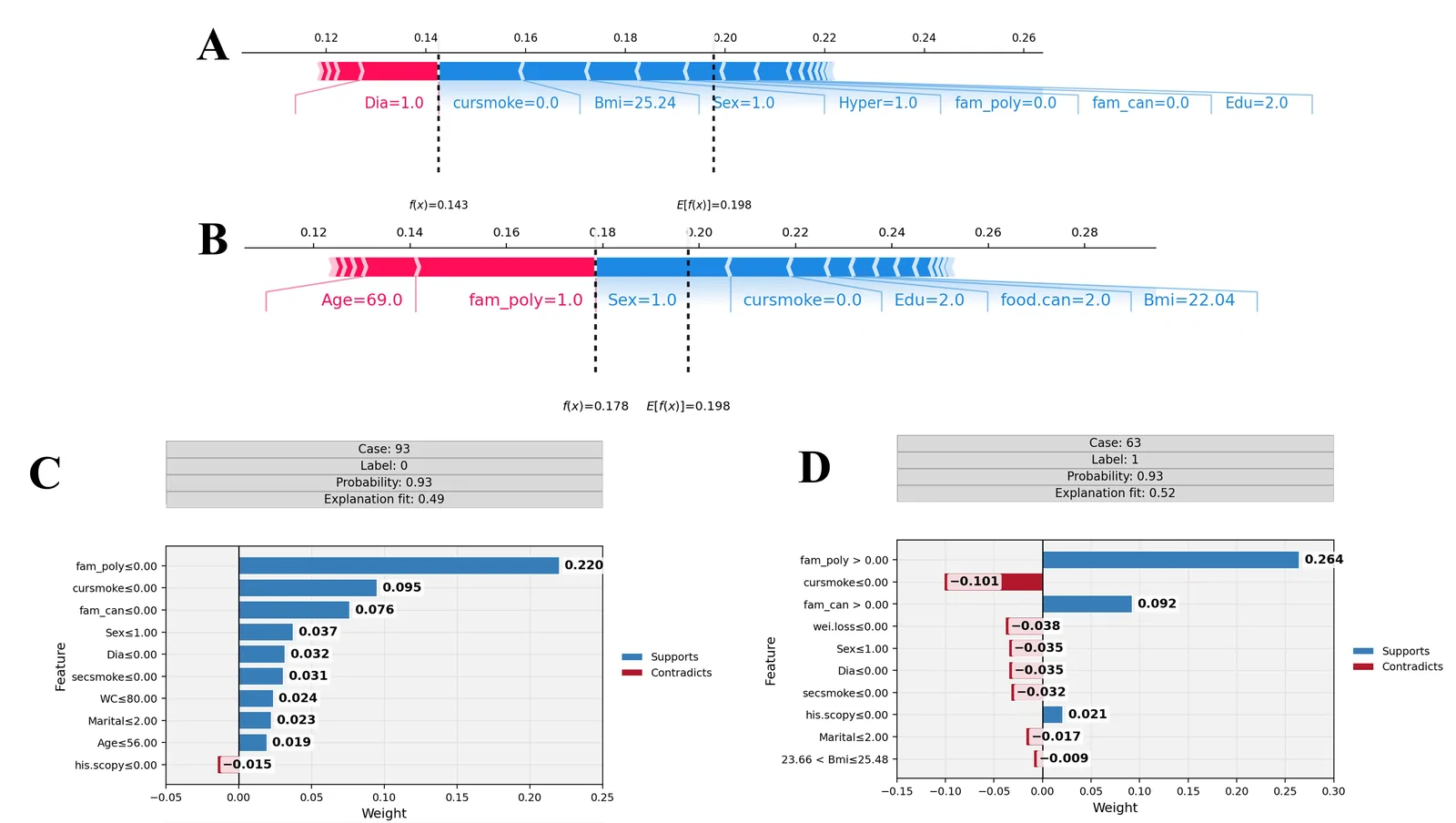
Individualized model interpretability via Shapley Additive Explanations (SHAP) method force plots and local interpretable model–agnostic explanations (LIME) local explanations. (A,B) SHAP force plots for a low-risk (*f*[*x*]=0.143) and a high-risk (*f*[*x*]=0.178) individual, where red and blue arrows represent risk-increasing and risk-decreasing effects, respectively. (C,D) LIME feature attribution plots for Case 93 (control) and Case 63 (case).

## Discussion

### Principal Findings

In this study, we developed and validated 9 ML models to enhance the initial risk stratification stage of the current population-based CRC screening framework. Using a large-scale screening dataset from Wenzhou, China, our results demonstrated that ML models, particularly the LightGBM algorithm, can effectively refine colorectal polyp risk assessment in individuals aged 50 to 74 years. By prioritizing a high recall (0.6503), our model ensures a robust “digital filter” that minimizes the risk of missed diagnoses during initial triage. Notably, our models achieved strong predictive utility by fully using the variables already collected during the initial screening phase. This suggests that the proposed ML approach can serve as a precision-driven triage tool, potentially improving the “yield” and cost-effectiveness of subsequent colonoscopies compared to traditional risk assessment methods.

Compared with previously published prescreening models, our LightGBM model showed substantially better discrimination. An electronic health record (EHR)–based AdaBoost model reported an AUC of 0.687 and sensitivity of 0.635, which is comparable to our results; however, such models are often limited by the incomplete capture of lifestyle and family history data in clinical databases [15]. A genetic risk score (GRS)–augmented LR model in a Caucasian cohort reached an AUC of 0.666, but required costly genotyping and had uncertain generalizability to Asian populations [33]. While some questionnaire- or nomogram-based models have reported AUCs ranging from 0.68 to 0.75, our model, achieving an AUC of 0.6724 and a superior sensitivity of 0.6503, offers a more streamlined approach by using only essential variables collected during the initial screening phase [34,35]. The robust performance of our LightGBM model is likely attributable to its ability to capture complex, nonlinear interactions and higher-order combinations among risk factors, such as the threshold effects of waist circumference and age identified in SHAP analysis. Unlike traditional LR and nomograms, this gradient-boosting framework effectively models these dependencies, providing a high-recall “digital triage” that is both cost-effective and specifically optimized for the East Asian population.

A distinguishing feature of our study is the deliberate prioritization of recall (0.6503) over precision in a screening context, where missing a polyp carries far greater clinical consequences than referring a low-risk individual for colonoscopy [36]. This recall is considerably higher than the 0.635 reported for the EHR-based model and provides a significant safety margin for population-level triage [15]. While our sensitivity does not yet reach the levels of intraprocedural computer-aided detection systems (0.90‐0.96), it is achieved noninvasively and at negligible cost using only baseline screening data [37-39]. Substantial studies have demonstrated that early detection of precancerous lesions through screening significantly reduces the burden of CRC [40,41]. By enabling targeted referral of truly high-risk individuals using a simple questionnaire that can be deployed at scale, our LightGBM model offers an efficient, equitable, and immediately implementable solution. This approach can dramatically improve the yield of limited colonoscopy resources, thereby maximizing the population-level impact of CRC screening programs in China and similar resource-constrained settings.

The clinical utility of our LightGBM model is underpinned by its capacity to prioritize and interpret multidimensional risk factors. SHAP analysis revealed that the model’s predictive power is primarily driven by current smoking, sex, and family history of colorectal polyps (FHP). Extensive evidence links cigarette smoking to an elevated risk of various polyp subtypes, including sessile serrated and hyperplastic polyps [42-44]. Notably, smoking cessation for over 20 years can reduce the risk to levels comparable to never-smokers [45], likely because tobacco-induced BRAF (B-Raf Proto-Oncogene, Serine/Threonine Kinase) mutations, a key mechanistic pathway, are primarily implicated in the serrated neoplasia sequence [46]. In terms of sex, males consistently have a higher prevalence of colorectal adenomas, advanced adenomas, and high-risk polyps than women in large screening cohorts [47,48]. Sex differences in colorectal polyp risk likely arise from interactions between sex steroids, body fat distribution, and adipokines, immune and inflammatory pathways, and sex-specific gut microbiota profiles [49,50]. Furthermore, FHP emerged as a critical predictor, a factor often absent or incomplete in prior models. Numerous studies confirm that FHP significantly increases the risk of colorectal polyps, particularly for early-onset cases or when multiple first-degree relatives are affected [51]. Inherited germline variants, such as mutations in DNA mismatch repair genes (eg, MSH3 and MLH3), are believed to underlie this predisposition [52]. Overall, the SHAP dependence plots (Figure 6B, D, and F) for these 3 factors also support the findings from most of the literature, in which males, current smokers, and those with a family history of colorectal polyps have the highest SHAP values.

While these categorical factors establish a significant risk baseline, the LightGBM model’s superior performance also stems from its ability to decipher complex, nonlinear effects within continuous clinical metrics. The following section details specific thresholds for BMI, waist circumference, and age, which provide a more granular risk profile than traditional linear assessments. Although BMI emerged as a stronger global predictor in our SHAP importance ranking, waist circumference provides critical localized insights into central obesity, which is often a more potent driver of colorectal neoplasia [53]. Notably, in Asian populations, individuals with a normal BMI but elevated waist circumference (90‐102 cm for men; 80‐88 cm for women) exhibit the highest risk of colorectal polyps [54]. This aligns closely with the nonlinear threshold of approximately 85 cm observed in our SHAP dependence analysis, a finding likely influenced by the predominance of female participants in our study sample. Similarly, BMI displayed a distinct nonlinear pattern, with a marked risk inflection point at 25 kg/m^2^. This threshold is consistent with international evidence from Australian, African American, and Iranian populations, where a BMI exceeding 25 kg/m^2^ consistently correlates with higher colorectal polyp prevalence [55-57]. The biological mechanisms for both indicators likely involve chronic inflammation and gut microbiota dysbiosis [58,59]. For instance, obesity-related inflammation can promote DNA mismatch repair defects and microsatellite instability, pathways particularly relevant to serrated polyps [60]. Furthermore, the higher abundance of *Fusobacterium nucleatum* in obese individuals is more strongly associated with serrated than with conventional pathways [61]. In addition to metabolic factors, age is a well-established primary risk factor for colorectal polyps and most chronic diseases. Epidemiological evidence consistently shows that the prevalence of colorectal polyps rises markedly after age 50, a pattern mirrored by the threshold at approximately 50 years in our SHAP analysis [62]. The underlying mechanism is thought to involve the progressive accumulation of somatic DNA mutations with aging, notably in the adenomatous polyposis coli gene, which initiates the classic adenoma-carcinoma sequence [63]. By integrating these nonlinear age and metabolic signatures, our model effectively captures the complex physiological transitions that define individual risk.

To bridge the gap between algorithmic prediction and clinical action, we used SHAP force plots as the primary tool for individual risk communication, supplemented by LIME for local validation. The force plot provides a transparent “risk balance sheet,” visualizing how specific predictors quantifiably push the individual’s risk score from the overall baseline toward a high-probability threshold. For instance, when triaging the high-risk individual in Figure 7B, the clinician can use the force plot to demonstrate that while certain factors might be protective, the combined “force” of being 69 years old and having a history of colorectal polyps necessitates an immediate colonoscopy. By replacing generic screening guidelines with this individualized, evidence-based visualization, our approach facilitates more nuanced patient-physician dialogue, builds clinical trust, and helps overcome patient reluctance in population-based settings.

### Strengths and Limitations

The strengths of our study are highlighted by several key aspects. First, our model was designed and validated within the context of a real-world, population-based screening program. Unlike studies relying on symptomatic hospital data, we exclusively used variables routinely collected during initial screening. This ensures that the tool is perfectly aligned with its intended deployment as a precolonoscopy triage mechanism. Second, we used a rigorous and reproducible modeling pipeline. Feature selection combined LASSO regression with the Boruta algorithm to balance interpretability and data-driven relevance. By using advanced ensemble algorithms like LightGBM, our approach effectively captured complex nonlinear interactions and clinical thresholds (eg, for age and BMI) that traditional LR cannot model, resulting in superior clinical utility. Third, this study addresses a critical clinical bottleneck: limited endoscopic resources. By prioritizing high recall, our model provides a resource-efficient strategy to ensure that truly “high-risk” individuals are fast-tracked for colonoscopy, thereby minimizing missed diagnoses and optimizing the efficiency of existing screening frameworks.

Despite these contributions, several limitations should be acknowledged. First, our models were developed using cross-sectional data from a single-center screening program in southeastern China, which may limit generalizability to other regions or ethnicities. Risk factor profiles vary geographically; for instance, dietary patterns and lifestyle stressors differ significantly between urban and rural populations [16,64]. External validation in diverse populations is therefore essential to confirm the robustness of our identified nonlinear thresholds, such as the BMI of 25 kg/m^2^ inflection point. Second, we did not stratify predictions by histopathological subtypes (eg, conventional adenomas vs sessile serrated lesions), which have distinct malignant potential. Future iterations using larger longitudinal datasets, aimed at specifically differentiating advanced neoplasia from nonadvanced lesions, are needed. Additionally, GRSs were not incorporated. While GRS can enhance predictive performance [33], our focus was on maintaining a zero-cost, high-accessibility tool for community-based triage. Lastly, although we provided interpretability through SHAP and LIME, the cross-sectional nature of the study precludes definitive causal inferences. Future longitudinal research is warranted to validate the biological trajectories identified by our ML framework.

### Conclusions

In conclusion, this study explored several ML models to enrich the current population-based CRC screening protocol. By using only noninvasive, routinely collected clinical and lifestyle variables, the XGBoost and LightGBM models achieved the best discriminative performance, while the latter excelled in recall, which is important in the screening context. These models function as a sophisticated “digital triage tool” that can be seamlessly integrated into the initial assessment phase of mass screening. By identifying truly high-risk individuals among asymptomatic populations, this approach supports a more efficient allocation of limited colonoscopy resources, potentially reducing the burden on health care systems. This study provides a scalable, data-driven solution to bridge the gap between initial risk assessment and definitive diagnostic intervention.

## Supplementary material

10.2196/89422 Multimedia Appendix 1 Number and histopathological classification of detected colorectal polyps.

10.2196/89422 Multimedia Appendix 2 The coefficient values of included features in the least absolute shrinkage and selection operator (LASSO) regression model.

10.2196/89422 Multimedia Appendix 3 Correlation matrix of 21 selected features.

10.2196/89422 Multimedia Appendix 4 Comparison of performance of selected machine learning models for colorectal polyps risk in the testing dataset.

10.2196/89422 Multimedia Appendix 5 Confusion matrix for colorectal polyp risk stratification models with different machine learning models in the testing dataset.

10.2196/89422 Multimedia Appendix 6 The calibration curve for colorectal polyp risk stratification models with different machine learning models in the testing dataset.

## Abbreviations

AP: average precision
APC: adenomatous polyposis coli
AUC: area under the curve
CatBoost: categorical boosting
CRC: colorectal cancer
GRS: genetic risk score
LASSO: least absolute shrinkage and selection operator
LightGBM: Light Gradient Boosting Machine
LIME: local interpretable model–agnostic explanations
LR: logistic regression
ML: machine learning
RF: random forest
SHAP: Shapley Additive Explanations
SMOTE: synthetic minority oversampling technique
XGBoost: Extreme Gradient Boosting
