# Supplementary material for: Development of an Interpretable Triage Tool for Colorectal Polyp Risk Stratification Within a Population-Based Screening Program: Machine Learning Approach
Source: JMIR Med Inform. 2026 Sep 30;14:e89422. doi: 10.2196/89422 (PMC13626646; doi:10.2196/89422)
Supplement: Supplementary file 1 [file medinform-v14-e89422-s001.docx]

Table S1. Number and histopathological classification of detected colorectal polyps.

| **Features** | | **Index (N=816)** |
| --- | --- | --- |
| **Number of Detected Polyps [n (%)]** | | |
|  | 1 | 677 (82.96%) |
|  | 2-4 | 133 (13.85%) |
|  | ≥ 5 | 26 (3.19%) |
| **Histopathological Classification [n (%)]** | | |
|  | Non-neoplastic Polyps | 530 (64.95%) |
|  | Serrated Lesions | 11 (1.35%) |
|  | Non-advanced Adenomas | 219 (26.84%) |
|  | Advanced Adenomas | 12 (1.47%) |
|  | Cancers | 44 (5.39%) |
