# Supplementary material for: Development of an Interpretable Triage Tool for Colorectal Polyp Risk Stratification Within a Population-Based Screening Program: Machine Learning Approach
Source: JMIR Med Inform. 2026 Sep 30;14:e89422. doi: 10.2196/89422 (PMC13626646; doi:10.2196/89422)
Supplement: Supplementary file 2 [file medinform-v14-e89422-s002.docx]

# **Table S2.** The coefficient values of included features in the lasso regression model.

| **Variables (names in model training)** | **Minimum criteria** | **1-SE criteria** |
| --- | --- | --- |
| Intercept | -4.251 | -1.596 |
| Body mass index (Bmi) | 0.035 | 0 |
| Waist Circumference (WC) | 0 | 0 |
| Age (Age) | 0.017 | 0 |
| Ethnicity (Ethics) | 0 | 0 |
| Sex (Gender) | 0.147 | 0 |
| Education Attainment (Edu) | 0.142 | 0 |
| Marital Status (Martial) | 0.081 | 0 |
| NSAIDs (Inflammed) | 0 | 0 |
| Anticoagulant (Clotmed) | 0 | 0 |
| Current smoker (cursmoke) | 0.880 | 0.685 |
| Secondhand smoking (secsmoke) | 0.249 | 0 |
| Alcohol Consumption (alcho) | -0.003 | 0 |
| Vegetables (food.veg) | 0 | 0 |
| Fruits (food.fruit) | 0 | 0 |
| Red meat (food.red) | 0 | 0 |
| White meat (food.white) | 0 | 0 |
| Legume (food.soy) | 0 | 0 |
| Pickled food (food.can) | -0.056 | 0 |
| Processed food (fooo.pro) | 0 | 0 |
| Fried food (food.fry) | 0.159 | 0 |
| Whole grain (food.whole) | 0 | 0 |
| Adequate physical activities (ad_rec_physic) | 0 | 0 |
| Family history of cancer (fam_can) | 0.317 | 0.061 |
| Family history of colorectal polyps (fam_poly) | 1.338 | 1.017 |
| Abdominal pain (ab.ache) | 0 | 0 |
| Abdominal distension (ab.dis) | 0 | 0 |
| Abdominal mass (ab.mass) | 0 | 0 |
| Abnormal defecation (bowel.ab) | 0 | 0 |
| Hematochezia (blood.stool) | -0.104 | 0 |
| Iron deficiency anemia (iron.animia) | 0 | 0 |
| Unexplained weight loss (wei.loss) | -0.695 | 0 |
| Hypertension (Hyper) | -0.078 | 0 |
| Hyperlipidemia (Hyperlip) | 0 | 0 |
| Diabetes (Dia) | 0.289 | 0 |
| Gout (Gout) | -0.044 | 0 |
| History of colonoscopy (his.scopy) | -0.300 | 0 |
