# Supplementary material for: Development of an Interpretable Triage Tool for Colorectal Polyp Risk Stratification Within a Population-Based Screening Program: Machine Learning Approach
Source: JMIR Med Inform. 2026 Sep 30;14:e89422. doi: 10.2196/89422 (PMC13626646; doi:10.2196/89422)
Supplement: Supplementary file 3 [file medinform-v14-e89422-s003.docx]

Note. There was no pair showing strong correlation (coefficient > 0.8). The color of the cells more close to blue means stronger positive correlation coefficient while more close to red indicates stronger negative correlation coefficient. Body mass index (Bmi); Waist Circumference (WC); Age (Age); Sex (Gender); Education Attainment (Edu); Marital Status (Martial); Current smoker (cursmoke); Secondhand smoking (secsmoke); Alcohol Consumption (alcho); Red meat (food.red); White meat (food.white); Pickled food (food.can); Fried food (food.fry); Family history of cancer (fam_can); Family history of colorectal polyps (fam_poly); Hematochezia (blood.stool); Iron deficiency anemia (iron.animia); Unexplained weight loss (wei.loss); Hypertension (Hyper); Diabetes (Dia); Gout (Gout); History of colonoscopy (his.scopy).
