# Supplementary material for: Development of an Interpretable Triage Tool for Colorectal Polyp Risk Stratification Within a Population-Based Screening Program: Machine Learning Approach
Source: JMIR Med Inform. 2026 Sep 30;14:e89422. doi: 10.2196/89422 (PMC13626646; doi:10.2196/89422)
Supplement: Supplementary file 4 [file medinform-v14-e89422-s004.docx]

Table S3. Comparison of performance of selected ML models for colorectal polyps risk in testing dataset.

| **Models** | **AUC [95%CI]** | **Accuracy** | **Recall** | **Specificity** | **PPV** | **NPV** | **F1 score** | **Brier score** | **Cutoff** |
| --- | --- | --- | --- | --- | --- | --- | --- | --- | --- |
| XGBoost | 0.672  [0.623, 0.722] | 0.7150 | 0.4663 | 0.7766 | 0.3408 | 0.8545 | 0.3938 | 0.1417 | 0.2432 |
| LightGBM | 0.664  [0.612, 0.713] | 0.5822 | 0.6503 | 0.5653 | 0.2704 | 0.8671 | 0.3820 | 0.1483 | 0.1395 |
| RF | 0.659  [0.609, 0.709] | 0.6334 | 0.5460 | 0.6550 | 0.2816 | 0.8535 | 0.3716 | 0.1407 | 0.1967 |
| LR | 0.657  [0.609, 0.708] | 0.6772 | 0.5031 | 0.7204 | 0.3083 | 0.8541 | 0.3823 | 0.1420 | 0.1996 |
| CatBoost | 0.652  [0.601, 0.704] | 0.6821 | 0.5092 | 0.7249 | 0.3144 | 0.8564 | 0.3888 | 0.1412 | 0.2016 |
| MLP | 0.644  [0.594, 0.696] | 0.6784 | 0.4724 | 0.7295 | 0.3020 | 0.8481 | 0.3684 | 0.1445 | 0.2083 |
| NB | 0.630  [0.579, 0.681] | 0.5920 | 0.5583 | 0.6003 | 0.2571 | 0.8458 | 0.3520 | 0.2547 | 0.2263 |
| DT | 0.629  [0.577, 0.679] | **0.7759** | 0.3374 | 0.8845 | 0.4198 | 0.8435 | 0.3741 | 0.1433 | 0.3120 |
| SVM | 0.607  [0.554, 0.661] | 0.7101 | 0.3926 | 0.7888 | 0.3153 | 0.8398 | 0.3497 | 0.1436 | 0.1843 |

**Notes:** ML: machine learning; AUC: area under the ROC curve, 95% confident interval was obtained with DeLong method; ROC: receiver operating characteristic; DT: decision tree; LR: logistic regression; SVM: support vector machine; RF: random forest; MLP: multilayer perceptron; NB: naïve bayes; Catboost: categorical boosting; XGboost: extreme gradient boosting; LightGBM: Light Gradient Boosting Machine; NPV: negative predictive value; PPV: positive predictive value.
