# Supplementary material for: Development of an Interpretable Triage Tool for Colorectal Polyp Risk Stratification Within a Population-Based Screening Program: Machine Learning Approach
Source: JMIR Med Inform. 2026 Sep 30;14:e89422. doi: 10.2196/89422 (PMC13626646; doi:10.2196/89422)
Supplement: Supplementary file 5 [file medinform-v14-e89422-s005.docx]

The confusion matrices illustrate the performance of nine machine learning models: DT, decision tree; LR, logistic regression; SVM, support vector machine; RF, random forest; MLP, multilayer perceptron; NB, naïve bayes; CatBoost, categorical boosting; XGBoost, extreme gradient boosting; and LightGBM, light gradient boosting machine. The rows represent the actual clinical diagnosis (gold standard), and the columns represent the model'’s predicted risk categories. Cells on the main diagonal (bottom-left and top-right) indicate correctly classified cases (true negatives and true positives), while off-diagonal cells (top-left and bottom-right) represent misclassifications (false positives and false negatives).
