# Supplementary material for: Development of an Interpretable Triage Tool for Colorectal Polyp Risk Stratification Within a Population-Based Screening Program: Machine Learning Approach
Source: JMIR Med Inform. 2026 Sep 30;14:e89422. doi: 10.2196/89422 (PMC13626646; doi:10.2196/89422)
Supplement: Supplementary file 6 [file medinform-v14-e89422-s006.docx]

The calibration curves illustrate the agreement between the predicted probability of colorectal polyps (x-axis) and the actual observed proportion (y-axis). The dashed 45-degree line represents a perfect calibration (the ideal model). Curves closer to this diagonal line indicate better model calibration. DT: decision tree; LR: logistic regression; SVM: support vector machine; RF: random forest; MLP: multilayer perceptron; NB: naïve bayes; Catboost: categorical boosting; XGboost: extreme gradient boosting; LightGBM: Light Gradient Boosting Machine.
